# Self-Controlled Perspective: Nurses' Perceived Social Undermining and Knowledge-Sharing Behavior in Public Hospitals

**DOI:** 10.1155/2023/4686379

**Published:** 2023-08-16

**Authors:** Muhammad Ali, Mubbsher Munawar Khan, Lin Xiong

**Affiliations:** ^1^School of Economics and Management, Dongguan University of Technology, Dongguan, China; ^2^School of Management, University of Science and Technology of China, Hefei, China; ^3^Hailey College of Commerce, University of the Punjab, Lahore, Pakistan

## Abstract

Nurses work in a high-risk and uncertain environment, which may lead to harmful social interactions resulting in self-regulation impairment. The aim of this study was to examine the effects of perceived social undermining and how and when this perception affects nurses' knowledge-sharing behavior. We developed a conceptual framework of self-regulation impairment in which nurses' perceived social undermining (from supervisors and coworkers) depletes self-control resources, dampening their knowledge-sharing behavior. We hypothesized social adaptability and resource management ability as self-regulating capacities that mitigate the impairment process. Results from a multisource and multiwave in the public hospitals provided support to our hypotheses. This study yielded significant findings with theoretical and practical implications that provide leads for future investigations in the field of healthcare research.

## 1. Introduction

Seven decades ago, [1] stated that others in an organization (coworkers) “do the most to make our lives sweet or sour.” Unsurprisingly, extensive research has been conducted to better understand how environmental factors influence (e.g., perceived social support [2] and perceived social undermining [3]) and contribute to the “sweetness” and “sourness” of our lives. Social undermining is a form of mistreatment categorized as “a perception of others' expressions of negative affect (e.g., anger), negative evaluations, and behaviors that hinder one's goal attainment” [4]. It creates an environment where stressful stimuli cause individuals to experience different strain reactions and has been linked to numerous well-being and health-related outcomes [5]. For instance, employee perceptions of social undermining have been linked to various stress-related outcomes, including poor mental health [6], psychological distress [7], turnover [8], sleep quality and work engagement [4], and deviant behavior [9]. In addition, Vinokur and Van Ryn [6] revealed that compared with social support, social undermining has an asymmetrically greater impact on employees' well-being and reactions in stressful situations.

Despite the well-established theoretical foundation of social undermining, studies on social undermining mostly emphasized its antecedents (e.g., [10–16]) and consequences (e.g., [4, 6, 8]). A limited number of studies have addressed the within-individual level factors that are competent for extenuating the destructive effects of perceived social undermining (e.g., [5]) on potential detrimental behavioral outcomes, especially in the healthcare service sector such as hospitals. The studies that have been conducted so far suggest that self-efficacy as an individual difference [17], trait resilience [4], social identification [18], and moral identity [19] can buffer the relationships between perceived social undermining and its consequences. To the best of our knowledge, these studies lack a single, cohesive explanation for within-individual mitigating effects.

Moreover, the detrimental impacts of perceived social undermining such as work engagement [4]; job attitudes, well-being, and deviant behavior [20]; revenge, avoidance, and reconciliation [21]; and engaging in undermining [22] on individuals have been studied. However, employee behavioral outcomes have been paid less attention [17]. Especially individuals' knowledge-sharing behavior is an imperative work outcome that continues to be understudied in social undermining literature. According to scholars, knowledge and its management are important for the competitive advantage, success, and long-term sustainability of organizations [23]. Given that knowledge sharing is one of the most important discretionary behaviors in the modern knowledge-based economy, it is critical to examine the conditions under which employees can or cannot do so [24, 25].

In this study, we incorporated these issues and developed a model that integrates a blended integrative resource regulation perspective (see [26] for more details) with the literature on perceived social undermining [27], knowledge sharing through self-control resource depletion, and within-individual buffering factors, namely, social adaptability and resource management ability. The aim of this study was to identify additional factors that mitigate the negative affective and behavioral reactions of individuals to perceive social undermining. Our research focuses on distinctions that highlight an individual's ability to exert a direct influence on threatening circumstances. Building on the resource regulation perspective, we argue that perceived social undermining (both from supervisors and coworkers) depletes individuals' self-control resources, which leads to dysfunctional behavior (knowledge sharing). Moreover, we further propose that employees with high levels of social adaptability and resource management ability will encounter fewer adverse reactions (i.e., self-control resource depletion) and have improved behavioral outcomes (i.e., knowledge sharing) when faced with social undermining from supervisors and coworkers than less adept employees. Given that social undermining is a perception and that it is possibly unrealistic to eliminate such sensitivities from individuals' cognitions, it is crucial to identify the factors that can mitigate its harmful effects.

This study makes several significant contributions. First, it contributes to the research on interpersonal relationships by demonstrating how social undermining shapes nurses' deviant behavior via self-control resource depletion. Second, by investigating the buffering roles of social adaptability and resource management ability in healthcare nursing departments, it provides a deeper comprehension of the relationship between the perceptions of social undermining and nurses' behavioral outcomes. Finally, from a blended integrative resource regulation perspective, we investigated how destructive behaviors (supervisors and coworkers undermining) affect nurses' willingness to share their knowledge, which has not yet been thoroughly investigated in previous studies on harmful social interactions [26, 28] but was recently recommended in a study on social undermining [4].

## 2. Theory and Research Framework

Social undermining at work refers to dysfunctional behavior [29] perceived as stressful both cognitively and emotionally [21, 30] and is anticipated to deplete individuals' self-control resources when viewed through the lens of self-regulation (e.g., [19]). As required by societal standards [31], individuals must suppress not only undermining cognitive and behavioral manifestations but also emotional manifestations [32]. Such suppression requires self-control, and exercising self-control uses up an individual's limited supply of resources for self-regulation [33]. In addition, employees' perceptions of undermining are psychologically taxing because they make them consider the causes and effects of maladaptive treatment [34]. As a result, self-regulation, or the capacity to restrain instinctive impulses and reactions, is necessary for appropriate responses to undermine threats [35, 36] and thus promote goal pursuit and context-appropriate behavior [37]. In our study, we propose the theory that the depletion of self-control resources serves as a mechanism to elucidate how social undermining perceptions influence employees' subsequent behaviors. Negatively valenced events consume more self-regulatory resources than positively valenced events, causing dysfunctional social encounters [27, 35, 37]. Previous research has linked social interpersonal problems to outcomes such as decreased prosocial behavior and increased deviance [38]. In fact, individuals often use self-regulatory mechanisms to restrain their urges and carefully consider the situation before acting when they are subjected to unfair treatment [34]. According to scholars (e.g., [35, 37]), this could result in energy depletion, which would then adversely affect individuals' other attitudes and behaviors [39]. Integrating a blended resource regulation perspective [26], we emphasize that when an individual's cognitive and physical resources are depleted, stress can occur, and this resource depletion further leads to stress-driven behavioral reactions and outcomes. Moreover, researchers have revealed that individuals' self-control resources are depleted by factors at work such as justice social comparison perception [40], emotional dissonance [41], family-work conflict [42], daily procedural and interpersonal justice behaviors [43], experienced incivility [44], ethical leader behavior [45], surface acting [46], contingent punishment [47], air pollution [48], and undermining victimization [22].

Concerning our review of the existing studies, we focused on acts of helping, which require self-regulatory resources [43]. When individuals' resources are depleted by social undermining, we expect them to engage in fewer helping behaviors (e.g., knowledge sharing) and more uncivil ones (e.g., increased counterproductive work behavior [48]). Our study used the self-regulation theory to better comprehend how individuals respond to the perceived social undermining of supervisors and coworkers on the basis of their characteristics. In the present study, we acknowledge that subordinates' social adaptability levels can significantly affect how they view their social interactions with supervisors and coworkers within an organization. Our research objective was to comprehend how social adaptability as a personal trait can affect individuals' responses to perceptions of social undermining. The ability to modify behaviors and adjust cognitions in response to shifting threats and situational demands is referred to as social adaptability [49]. According to Ployhart and Bliese [50], “It is [the] underlying characteristics of an individual that represents his or her ability, skill, disposition, willingness, and/or motivation to change or fit the different task, social, and environmental features” (p. 13).

**Figure 1:**
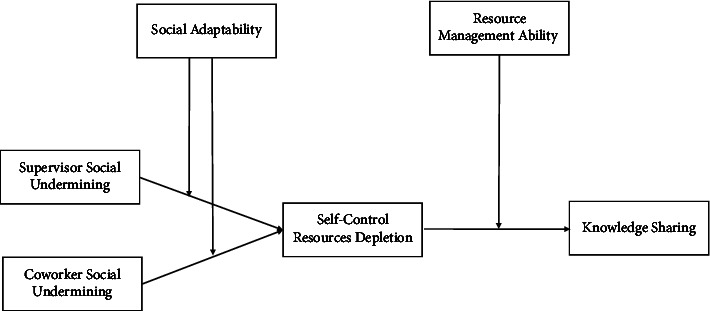
Proposed conceptual model.

In accordance with the integrative resource regulation perspective, resource management ability (i.e., individual differences in control beliefs) was investigated as a factor for mitigating the negative effects of mistreatment [51]. The construct of resource management ability was developed by Hochwarter et al. [52, 53] and refers to “one's ability to maintain and mobilize one's resources for personal benefit.” According to Bolger and Zuckerman's [54] differential exposure-reactivity model, individual differences can affect how employees see stimuli as stressors and how they react to them, which fits well with the adopted theoretical perspective. In our study, we propose the buffering role of social adaptability (first-stage moderator) between the relationship of perceived undermining and self-control resource depletion and that of resource management ability (second-stage moderator) between the relationship of self-control resource depletion and knowledge behavior, which are posited to influence individuals in the workplace from a blended integrative resource regulation perspective. Consequently, we infer that individuals with better social adaptability and perceived resource management ability will exhibit more positive outcomes (i.e., self-control resource) and behavioral reactions (i.e., knowledge sharing) in response to perceived undermining than those who report low levels of social adaptability and resource management ability.  Figure 1 depicts the conceptual model of the study.

## 3. Hypotheses Development

### 3.1. Social Undermining and Self-Control Resource Depletion

According to existing resource depletion models [35, 37], social undermining impairs an individual's self-control resources [19]. It is recognized as a hindrance stressor with several negative consequences for organizations and individuals [17]. Furthermore, it is a destructive work behavior of coworkers and supervisors that refers to “the negative form of social interaction characterized by active dislike and devaluing of an individual” [8, 55]. A point of agreement among undermining scholars is that perceiving undermining not only affects the recipient's moods, hedonic tone, and proactive socialization [8] but also harms [6] and entails a deliberate set of actions that would worsen the recipient's circumstances than what they otherwise would be [27]. As previously indicated, workplaces are frequently riddled with a plethora of stressors that necessitate individuals to exercise self-regulation [56–58]. The theory of self-regulation is a comprehensive theory that describes how individuals regulate their emotions, motivation, behaviors, and cognition to accomplish their objectives [35]. These resources are finite [59, 60], and interpersonal aggression is more likely to occur in cases of depletion because the individual lacks the self-control needed to restrain aggressive impulses [42]. As a result, individuals' psychological, mental, and physical resources (i.e., self-regulatory) are critical in assisting them in combating aggression and maintaining the effort. These resources can be depleted when individuals are involved in acts of self-regulation [61], reducing their ability to self-regulate [37], which leads to psychological strain [39]. Furthermore, academics have argued that being the target of intrapersonal aggression at work creates a conflict between the desire to take revenge and the higher-order objectives of acting by interpersonal norms or maintaining good relationships, which depletes self-control resources [33, 44].

  H1: supervisors' social undermining and coworkers' social undermining are positively associated with self-control resource depletion.

### 3.2. Self-Control Resource Depletion as a Mediating Mechanism

According to Hypothesis 1, social undermining has a direct impact on individuals' self-control resource depletion. In this study, we examined how this impact may further affect individuals' knowledge-sharing behavior. Knowledge is regarded as a competitive advantage for organizations, and for success and long-term sustainability, management of knowledge is critical [62]. On the other hand, scholars are increasingly realizing that employees, rather than technologies or even systems, are the main obstacles to knowledge management processes [63]. Even though knowledge sharing is one of the most important discretionary behaviors [64], it is significant to examine the circumstances under which individuals may or may not share their knowledge in today's advanced knowledge-based economy.

To identify potential behavioral outcomes, we employed a resource regulation perspective in conjunction with the literature on mistreatment [4, 26]. Existing research indicates that destructive social behavior (i.e., social undermining) may result in both deviant and prosocial behaviors (e.g., [65]), consistent with a recent study on the impact of resources for self-regulation on discretionary behaviors (e.g., [4, 66]). To achieve goals such as being helpful or abstaining from negative behavior, self-control is essential [67, 68]. However, employees are more likely to experience breakdowns in their capacity to control their subsequent behavior while at work because undermining depletes resources for self-regulation [43]. The consequences of resource depletion are frequently obvious as low-intensity, ambiguous, non-role-prescribed, and deviant behaviors [69], as this represents a burden that consumes regulatory resources that could instead be used by individuals at work [40, 70]. Therefore, resource-drained employees may engage in fewer sharing behaviors to protect the limited attentional resources that they still have instead of taking on more work that benefits others [40, 71]. Employees might not be as motivated to invest their remaining resources in sharing behavior [72] because it is unrelated to their official job duties [69]. Therefore, we propose that individuals who encounter social undermining may exhaust their reserves of self-control, further weakening self-regulation, reducing their ability to control their aggressive behaviors, and increasing their propensity to act destructively.

This is because knowledge sharing necessitates more costs or risks from individuals than other discretionary behaviors. It involves the sharing of special skills, information, expertise, and specialized knowledge, and individuals may require additional effort and time to engage in such behaviors. In addition, individuals within the organization might decide not to divulge insider information to others to keep their competitive advantages [63]. Thus, encouraging knowledge sharing can be particularly difficult unless individuals think doing so will have greater advantages [25, 73]. Consequently, it is critical to identify the factors that may prevent knowledge sharing among organizational members [74].

Typically, the presence of a stressor causes self-regulation impairment (e.g., social undermining) that depletes an individual's self-control resource [4], reducing the ability to control impulses and exhibit socially desirable behaviors [75]. Supervisors and coworkers are essential to an employee's relationships, work-related achievements, and reputation in the workplace; consequently, employees have strong reactions to undermining from these individuals (e.g., [27, 76]).

  H2: supervisors' social undermining and coworkers' social undermining have negative indirect effects on knowledge-sharing behavior through self-control resource depletion, such that social undermining increases resource depletion, which then decreases knowledge-sharing behavior.

### 3.3. Social Adaptability as a Buffering Mechanism

Employees' perceptions of social problems (e.g., undermining) have a negative impact by taxing self-regulation processes and depleting the necessary self-control resources [36]. Employees' abilities to regulate their behaviors are debilitated as self-control resources are depleted. Individuals, rather than being passive bearers of threatening events, must constantly self-regulate to control the negative consequences of these events [33, 77]. Thus, it is likely that individual differences in the ability for self-control influence the negative impact of perceived social undermining on knowledge-sharing behavior by compromising self-control resources. Among the potential individual differences in self-regulation capacity, social adaptability plays a key role in the anti-stress response process generally (e.g., [26, 78]) and in the stressful nursing context specifically [79]. We propose that individual social adaptability (e.g., as a personal characteristic) serves as a stress-relieving resource for individuals at work when they perceive undermining from supervisors and coworkers. Theoretical underpinnings indicate that social adaptability provides the basis for examining how resources enable individuals to perceive and react to their surroundings. To respond, process, and comprehend social undermining (supervisors and coworkers), highly adaptable individuals may use social adaptability as a resource to reduce their need for self-regulation. Individuals' natural homeostatic balances are less likely to be disturbed when highly adaptable individuals' self-regulatory responses are less resource demanding [80].

As previously reported, perceived social undermining behaviors are insidious ([27], p. 332). Despite the veracity of previously confirmed relationships [20, 26], we propose that social adaptability can be a buffering influence of social undermining on behavioral outcomes through self-control resource depletion. By integrating a resource regulation perspective, we propose the effects of perceived social undermining as a function of variations in individual social adaptability, which is similar to Ployhart and Bliese's [50] conceptualization. We specifically contend that individuals who lack social adaptability are more likely to experience the adverse effects of stressful situations with undermining contexts because they lack or have exhausted their resources for self-control and are unable to control their behavior. By contrast, individuals with high social adaptability have the means and tools to control any potential negative effects of situations where social undermining behavior is perceived.

  H3: social adaptability buffers the indirect relationship between supervisors' and coworkers' social undermining (via self-control resource depletion) and knowledge sharing, such that the mediated relationship is weaker when an individual has high (vs. low) social adaptability.

### 3.4. Resource Management Ability as a Buffering Mechanism for Social Undermining Effects

Individuals with more resources in an organization are better able to control their behaviors in response to workplace stress and successfully manage them (e.g., [81]). Integrating the resource regulation perspective, we posit that individuals' perceived undermining from supervisors and coworkers may interrupt the restoration of their self-regulatory resources, as evidenced by depleting self-control resources, which further affects their knowledge-sharing behavior. We hypothesized that such relationships depend on an individual's resource management ability. Although the arguments of the conservation of resources (COR) theory and resource-based self-regulation theory are conceptually similar and share many other similarities (see [82] for details), Halbesleben et al. [83] claimed that the self-regulation theory provides a potential lens to investigate the loss process, arguing that COR is less clear in the process of losing resources. Therefore, to clarify loss as a self-regulation impairment process, we opted for a blended integrative resource regulation perspective [26].

The resource regulation theory identifies resource management ability (i.e., “equipped to protect and acquire resources that include access to equipment, assistance, flexibility, and control over the pace of, and exertion towards, one's work” [81]) as a key source of employees' coping efforts [51]. Resource management ability is a unique characteristic that has been shown to improve health and well-being, especially in high-stress situations [81]. According to scholars (e.g., [84, 85]), the ability to manage resources is likely to offer affective and cognitive resources that make up for exhausting circumstances. Specifically, higher levels of employee resource management ability buffered the harmful effects of mistreatment on employee-reported behavioral outcomes [51, 81]. Among others, they promote effective coping with such stress-inducing demands because they provide individuals with a sense of self-control [52]. According to Halbesleben et al. [83], it is significant that an individual's ability to manage resources goes beyond the mere possession of resources by emphasizing an individual's capacity to make the best use of already-existing resources or acquire new resources that can protect against job stressors.

We argue that employees with high levels of resource management ability may be less susceptible to the damaging effects of self-control resource depletion on knowledge-sharing behavior because employee resources enable them to capably counterbalance demands. Empirical studies also recommend that resource management ability counters the depleting effects of stressors on employees' affective, cognitive, and behavioral work outcomes [51]. According to Frieder et al. [81], individuals with high levels of resource management ability showed less emotional exhaustion, intention to quit, dissatisfaction, and decreases in work effort when dealing with mistreatment behavior. However, little attention has been given to examine how within-individual differences in individual characteristics may moderate the extent to which depleted self-control resources influence individual knowledge-sharing behavior. This oversight is regrettable for practical reasons because individual characteristics can be easily changed to help employees. If greater resource management ability provides individuals with resources to combat resource depletion, then organizations can address the issues caused by undermining behaviors. Integrating the resource regulation theory, we propose that perceived social undermining can interfere with the recovery of self-regulatory resources, resulting in impaired self-control, which affects behavioral outcomes (individual knowledge-sharing behavior). Therefore, we hypothesized that such relationships depend on individuals' resource management ability. The following hypothesis is proposed on the basis of the preceding discussion:

  H4: resource management ability buffers the indirect relationship between supervisors' and coworkers social undermining (via self-control resource depletion) and knowledge sharing, such that the mediated relationship is weaker when an individual has high (vs. low) resource management ability.

## 4. Methods

### 4.1. Participants and Procedures

The respondents were nurses working at public hospitals in Lahore, Pakistan. We used a three-wave survey structure, with a 3-week interval between each wave, to control for common method bias [86] and followed the process described in our model. The respondents were assured of the confidentiality of their data and asked to place their completed surveys in envelopes, which they sealed and returned to the data collection team.

The distribution procedure for the survey packets to head-nurse and subordinate-nurse pairs was outlined in guidelines and communicated to our data collection team and administrative department. We asked the administrative personnel to compile a list of head-nurse and subordinate-nurse pairs before conducting the survey. Head nurses can easily obtain information about the behavior of their subordinate nurses because hospitals typically operate as teams with relatively active formal and informal communication among staff members. Head nurses who had the chance to observe the knowledge-sharing behaviors of their immediate subordinate nurses were given a rating form. Thus, the likelihood of self-selection bias is probably low.

At time 1, we collected information related to demographics, perceived social undermining (head-nurses and subordinate nurses), and social adaptability. At time 2, we gathered data on self-control resource depletion and resource management ability, and at time 3, the head nurses were asked to respond to questions pertaining to the demographics and knowledge-sharing behavior of their subordinates. Codes were used to match the responses of the head and subordinate nurses. While not too long to make it likely that significant internal or external events occurred during the data collection, these time intervals were sufficient to alleviate concerns regarding reverse causality. The questionnaires were administered in English, which is regarded as an official language in Pakistan [87].

Initially, 650 head-subordinate nurse dyads were invited to participate in the survey, and 495 responses were received in three waves. After removing incomplete and mismatched responses, a final sample of 440 matched responses was collected, with a response rate of 67%. More than half (75.2%) of the respondents were female. The participants' mean (SD) age was 28.7 (4.01) years, and their mean (SD) tenure was 2.19 (1.19) years.

## 5. Measurement

### 5.1. Perceived Social Undermining

Perceived social undermining was assessed using measures from the study of Duffy et al. [27] which were widely used in earlier studies (e.g., [10, 17, 20]). The participants were requested to rate their supervisors' and coworkers' undermining behaviors, for example, whether they had behaved in ways that insulted them and hurt their feelings over the past week, on the basis of a 5-point Likert scale (from 1 = to no extent to 5 = to a great extent). The Cronbach alpha value was 0.98.

### 5.2. Social Adaptability

To measure social adaptability, a five-item scale was adopted from Baron and Markman [88] which was validated by Mackey et al. [26]. Nurses were asked to respond from 1 (strongly disagree) to 5 (strongly agree). A sample item is “I can easily adjust to being in just about any social situation.” The Cronbach alpha value was 0.94.

### 5.3. Resource Management Ability

To measure resource management ability, six items were adapted from Hochwarter et al. [52] which have been used in previous studies [51, 81]. A sample item is “I am able to pace myself at work when things get hectic.” The nurses were asked to respond on the basis of a 5-point Likert scale (from 1 = strongly disagree to 5 = strongly agree). A sample item is “When work is stressful, I am able to conserve my energy.” The Cronbach alpha value was 0.85.

### 5.4. Self-Control Resource Depletion

The five-item scale used to measure the depletion of individual self-control resources was adapted from Johnson et al. [42] which was originally developed by Twenge et al. [89] and previously used in the self-control literature [46, 48, 90]. The item included “I felt drained.” The Cronbach alpha value was 0.89.

### 5.5. Knowledge Sharing

Using a seven-item scale, immediate supervisors (head-nurses) assessed their subordinates' knowledge-sharing behavior, as adopted from Lee et al. [24]. A sample item is “The subordinate shares his/her special knowledge and expertise with others.” The Cronbach alpha value was 0.94.

### 5.6. Control Variables

Similar to previous studies [4, 46, 48], this study controlled for several characteristics of the respondents, including age, gender, job tenure, and negative affect. Age and gender are significant demographic factors that can influence the respondents' behaviors [48, 91]. They might also impact the assessments of perceived undermining. For instance, females might be more vulnerable to its negative effects. Given the unfavorable nature of interpersonal interactions, it makes sense to assume that socially negative appraisals affect people's negative emotions. However, in line with the resource-based approach to self-control, we argued that the depletion of self-control resources will specifically mediate the effects of socially undermining appraisals on individual behaviors [77]. To support our claims, we controlled for negative affect by using a 10-item scale adapted from the Positive and Negative Affect Schedule [92]. The Cronbach alpha value was 0.93.

### 5.7. Common Method Bias

Confirmatory factor analyses of the study variables were performed using the AMOS software package [93]. The six-factor baseline model showed an acceptable fit (*χ*^2^ = 2043.714, degrees of freedom (d*f*) = 1006, *χ*^2^/d*f* = 2.03, Tucker–Lewis index (TLI) = 0.95, comparative fit index (CFI) = 0.96, and root mean square error of approximation (RMSEA) = 0.04) compared with the one-factor model (*χ*^2^ = 8192.848, d*f* = 1021, *χ*^2^/d*f* = 8.02, TLI = 0.70, CFI = 0.73, and RMSEA = 0.12).  Table 1 shows the other comparison model. The results demonstrate no common method bias and verified that the constructs were sufficiently distinct. These results increase confidence that common method bias (CMB) is not a likely contaminant of the results observed in this study and that our measures are sufficiently free of overlap and consistent with previous studies [94–96].

## 6. Results

Table 2 shows the means, standard deviations, reliabilities, and intercorrelations among the study's variables. The unstandardized coefficients for the paths estimated in the model are shown in Table 3. Perceived social undermining (from supervisors and coworkers) was a significant predictor of individuals' self-control resource depletions (*β* = 0.300, 0.285, *p*  <  0.001, respectively), thus supporting our Hypothesis 1. To test the indirect effect between individuals' perceived social undermining and knowledge sharing, we applied PROCESS macro model 4. As shown in Table 4, the negative indirect effect is significant (*β* = −0.043, −0.040; SE = 0.20, 0.19; and 95% CI = not crossing zero), and supporting our Hypothesis 2.

**Figure 2:**
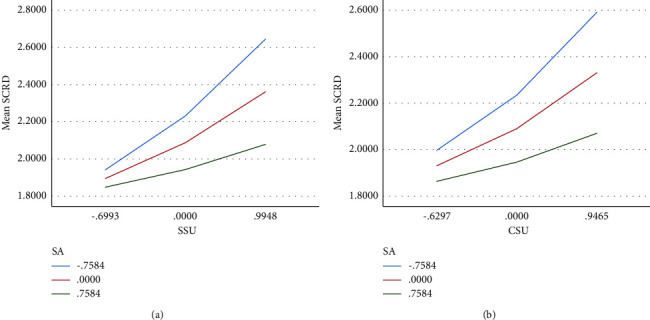
(a, b) The moderating effects of social adaptability.

Hypothesis 3 (first-stage moderated mediation model) posits that the indirect relationship between nurses' perceived social undermining (supervisors and coworkers) and individuals' knowledge-sharing behavior through self-control resource depletion is weak when an individual has high social adaptability. PROCESS model 7 with 5,000 bootstrap iterations was applied to test the interaction effect of undermining adaptability on individuals' self-control resource depletion. In particular, we computed the simple slopes for social undermining in predicting self-control resource depletion when social undermining was high vs. low (i.e., ±1 SD [97]). Results yielded that social adaptability was a significant moderator of the within-individual relationship between undermining and self-control resource depletion (supervisors undermining: *β* = −0.185, SE = 0.044, and 95% CI = not crossing zero and coworkers undermining: *β* = −0.162, SE = 0.048, and 95% CI = not crossing zero). Simple slope analyses showed that the relationship between social undermining and self-control resource depletion was significantly weaker among individuals with high social adaptability (supervisors undermining: *β* = −0.020, SE = 0.012, and not crossing zero and coworkers undermining: *β* = −0.019, SE = 0.013, and not crossing zero) than among those with low social adaptability (see Table 4 and Figures 2(a) and 2(b)). The moderated mediation index provides a formal test of moderated mediation [98] and produced significant results in this study (supervisors undermining index = 0.027 and coworkers undermining index = 0.024; not crossing zero), thereby demonstrating support for Hypothesis 3.

**Figure 3:**
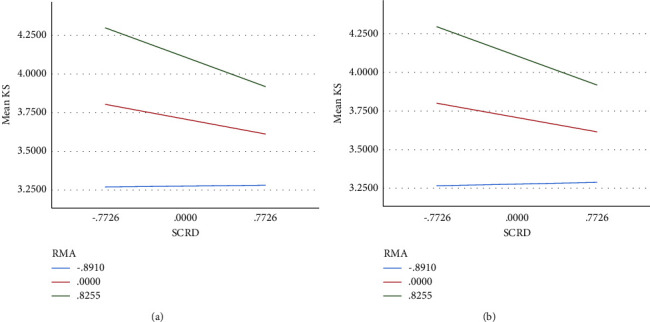
(a, b) The moderating effects of resource management ability.

Hypothesis 4 (second-stage moderated mediation model) predicted that the indirect effect of social undermining (via self-control resources depletion) on knowledge sharing would be weakened by high resource management ability. Following the same procedure as Hypothesis 3, we applied PROCESS macro model 14 and tested the effect of self-control resource depletion and resource management ability on individual knowledge-sharing behavior. Results confirmed that resource management ability was a significant moderator of the within-individual relationship between self-control resource depletion and knowledge sharing (supervisors undermining: *β* = −0.148 and SE = 0.049 and coworkers undermining: *β* = −0.151, SE = 0.049, and not crossing zero). Simple slope analyses further confirmed that the relationship between self-control resources depletion and knowledge sharing was significantly weaker among individuals having high resource management ability (supervisors undermining *β* = −0.071; SE = 0.023; and coworkers undermining *β* = −0.067, SE = 0.023, and not crossing zero, respectively); than those having low resource management ability (see Table 3 and Figures 3(a) and 3(b)). The moderated mediation index also produced significant results (index = −0.043 and −0.041, not crossing zero, respectively), thereby demonstrating support for Hypothesis 4.

## 7. Discussion

Individual knowledge-sharing behavior is important for organizational sustainable competitive advantages and effectiveness in the knowledge-based environment [24, 99]. Social interaction has a significant impact on how much individuals value their resources and how much effort they put forth to share their knowledge. This influence can either increase or decrease over time. This study used a resource-based self-regulation theory to examine how social undermining affects individual knowledge-sharing behaviors. We hypothesized and found that the social undermining perception experience explains dysfunctional outcomes. A focus on the detrimental aspects of social undermining provokes threat assessments, resulting in a taxing experience that depletes self-control resources and renders individuals less capable of controlling their behavior through proper social norms. Social adaptability and resource management ability are influential characteristics in this undermining (stress) experience because they create a lens by which individuals cope with social undermining. This study also examined the intervening effect of self-control resource depletion between social undermining and knowledge sharing, moderated by social adaptability and resource management ability. Individuals experience self-control resource depletion as perceived undermining increases, which reduces their knowledge-sharing behavior. Moreover, this mechanism is moderated by social adaptability and resource management ability. When social adaptability is high, the negative impact of social undermining on knowledge sharing through the depletion of self-control resources is mitigated.

### 7.1. Theoretical Implications

Individual knowledge sharing is becoming increasingly important in knowledge-based organizational environments [25]. Our findings point to several important insights that can provide a new, more complete explanation of knowledge sharing and social undermining.

In this study, we examined the important predictors of knowledge sharing, a key discretionary behavior in organizations [24]. First, we demonstrate that perceived social undermining hinders knowledge-sharing behavior. In a knowledge-based environment, it is important to identify factors that prevent knowledge sharing. From the blended integrative resource regulation perspective, our research confirms that when individuals experience undermining, they frequently use self-regulatory mechanisms first to restrain their urges and rationally comprehend the situation before directly engaging in retaliatory acts. This may deplete their self-control resources [61], negatively influencing their attitudes [40] and knowledge-sharing behavior [25]. We argued that to share their specialized knowledge, skills, or expertise, nurses may need to devote a fair amount of time and effort, as sharing behaviors involve actions and are likely to be complicated, over which individuals have some discretion. In light of this finding, encouraging knowledge sharing is more challenging than encouraging other optional behaviors such as organizational citizenship behavior.

Second, to investigate and expand our understanding of individual-situation interactions in the self-regulation impairment process [38], we examined the roles of social adaptability and resource management ability in assisting nurses to maintain physical and psychological functioning under interpersonal stress. Our findings support that social adaptability (first-stage moderator) and resource management ability (second-stage moderator) are protective factors that help buffer against self-regulation impairment and respond to the call of Fehr et al. [48] to examine the moderating factors (in the first and second stage) that buffer the effects of self-control resource depletion. This buffering effect occurs by weakening the stressor (i.e., perceived social undermining), strain (i.e., self-control resource depletion), and outcome relationship (i.e., knowledge sharing). According to Hypotheses 3 and 4, social adaptability lessens the stress reactions to initial stressors, whereas self-management skills make it easier to recover from the psychological strain that has already occurred. Although depletion makes it more difficult for employees to refrain from deviant and unethical behaviors (e.g., [34, 90]), individuals' self-management abilities are considered coping efforts [51] that enable them to override impulses, align behavior with social norms [77, 82], and buffer the harmful effects of mistreatment, which help improve their discretionary behaviors [51].

Third, our study contributes to the literature on individual self-control by identifying the important but overlooked antecedent (i.e., social undermining) appraisals of individual self-control resource depletion that are driven by an interrelated set of psychological factors. Research on the organizational implications of self-control has focused on a narrow range of predictors, usually job demands. Some researchers have theorized that a broader range of phenomena may cause employees to feel depleted, but empirical research on the links between self-control and broader contextual factors is limited. Our findings are consistent with those of recent studies that self-control resources are significant to individuals' daily lives [42, 58, 100].

Fourth, our results enhance the understanding of undermined behavioral relationships by integrating the COR and self-regulation theories. Most existing studies have used these theories separately on the basis of mistreatment literature, and limited studies have opted to use a blended integrative resource regulation perspective [26]. Our findings demonstrate that individual perceptions of social undermining cause self-control resource depletion and result in non-discretionary behaviors. However, their within-individual tactics are likely defensive instead of assertive [101]. Thus, as a remedy, they symbolize individual differences that are contextually engaging [102] and characteristically proactive [50]. From a blended theoretical perspective, we contend that when examined in the context of social undermining, social adaptability and resource management ability are inextricably linked and have assimilative considerations.

The innovative contribution of our study is the development of a theoretical framework from a resource regulation perspective and the nuanced explanation it offers as to how and when the perception of social undermining impairs nurses' helping behaviors. In contrast to previous theories of social undermining, the theory that we propose states that the process leading from social undermining to knowledge sharing is partly dependent on individuals' willingness to disable self-sanctions against harm doing. To our knowledge, no prior theories, particularly in nursing studies, have included self-control resource depletion as a mediating mechanism in the undermining and behavioral response relationship. However, we explore this model even further by proposing that social adaptability (stage-one moderator) and resource management ability (stage-two moderator) can buffer resource impairment and moral disengagement (knowledge sharing) as a response to social undermining. The explanation of how perceived social undermining leads to deviant behaviors involves multiple stages in which social-contextual factors can reinforce or weaken the key mediating mechanism (i.e., self-control resource depletion).

### 7.2. Practical Implications

The effects of perceived social undermining on individuals' self-control resources and knowledge sharing suggest several important management practices. First, our research shows that perceived social undermining is a significant stressor for individuals, reducing their self-control resources and leading to deviant behaviors. Even though hospitals invest in knowledge-management systems, they may not benefit from it if supervisors and staff undermine individuals. Therefore, organizations and managers should concentrate their time and energy on preventing social undermining and increasing awareness of harmful interpersonal interactions by implementing appropriate strategies. For instance, creating and offering coaching sessions or training programs to teach interpersonal relationship skills could help prevent social undermining. In addition, employing techniques for teamwork and conflict resolution in the workplace can help lessen instances of undermining [4].

Second, our results show that perceived social undermining depletes self-control resources and leads to deviant behavior. Therefore, it is necessary to find strategies to alleviate the detrimental effects of social undermining, as individuals' resources are limited [42, 59]. Our research reveals that social adaptability mitigates the negative effects of social undermining. Therefore, organizations and managers should pay more attention to employees' social adaptability and provide an environment that boosts healthy personnel or social interactions [6, 8]. Consequently, it is helpful for individuals to employ social practices that break the adverse links of undermining. Individuals with high social adaptability are not less affected by undermining, but their social adaptability is important for their discretionary behavior, that is, knowledge-sharing behavior [25, 103].

Third, although depletion makes it difficult to motivate individuals to engage in helping behavior, such effect can be mitigated. Moreover, owing to the extent that self-control resource depletion is an important underlying driver of deviant behaviors, individuals experiencing depletion have difficulty engaging in helping behaviors. Our study suggests that such effects can also be buffered by replenishing individuals' self-control resources. For instance, organizations can help employees regain their self-control resources by organizing self-affirmation training sessions to enable them to replenish depleted resources [46, 104]. Organizations can also help individuals conserve and regain resources and their resource management ability by providing counseling sessions (i.e., employee assistance programs [51] and through expressive writing interventions (see [105] for details). These research streams suggest a variety of interventions to reduce undermining in organizations.

### 7.3. Limitations and Future Directions

This study has several limitations. First, the authors relied on self-reported measures of perceived social undermining, self-control resource depletion, social adaptability, and resource management ability, which might have restricted our ability to objectively analyze social undermining activities, personnel characteristics, and resource impairment issues. Nevertheless, the self-reported characteristics of social undermining suit our research, as perceptions determine individual reactions [106]. In addition, previous studies have provided strong evidence that self-reported self-control practices yield accurate and trustworthy assessments of resource depletion (e.g., [68, 90]), social adaptability [26], and resource management ability [81]. Future studies are encouraged to employ objective metrics and solicit input from other groups of people.

Second, we measured social undermining from the same perspective as that in two undermining sources (head-nurse and subordinate-nurse) at the same time. Although our measurement followed the precedence of social undermining literature [27], the role of context was absent in this measurement. In addition, only head nurses reported knowledge-sharing behavior, which may not be the only reliable source of data because undermining head nurses reported the knowledge-sharing behavior of their direct subordinate nurses. Future research should collect data at different time waves and others' perspectives within-outside the organization (e.g., coworkers, patients, family members, and friends) and coworkers' perspectives regarding knowledge-sharing behavior.

Third, while we examined social adaptability and resource management ability as factors that influence within-individual interactions, future studies may expand our theoretical reasoning by adding other within- and between-personal factors. For example, sleep quality and quantity, job control, and trait resilience are important individual factors for coping with stressors [4, 68].

Fourth, our conceptual model was limited to two moderating variables, an independent variable, a mediating variable, and a dependent variable. Future research may consider a broad model with more than one variable that could describe the detailed underlying mechanism that affects the perceptions of and response to social undermining (the transactional model of stress [107]).

In addition, our research sample was restricted to head nurses and their immediate subordinates in hospitals within a single cultural context. Thus, the generalizability of the results of our study to other healthcare organizations in Pakistan and other cultural contexts might be limited.

## Figures and Tables

**Table 1:** Model comparison.

Model	Structure	*χ * ^2^	d*f*	Δ*χ*^2^ (Δd*f*)	CFI	TLI	RMSEA
Baseline model	Six-factor	2043.714	1006		0.96	0.95	0.04
Model 1	Five-factor	3430.321	1011	1386.607 (5)	0.91	0.89	0.07
PSU and PCU							
Model 2	Four-factor	5056.005	1015	1625.684 (4)	0.84	0.83	0.09
PSU, PCU, and SA							
Model 3	Three-factor	6172.611	1018	1116.606 (3)	0.80	0.78	0.10
PSU, PCU, SA, and SCRD							
Model 4	Two-factor	7000.920	1020	828.309 (2)	0.77	0.75	0.11
PSU, PCU, SA, SCRD, and RMA							
Model 5	One-factor	8192.848	1021	1191.928 (1)	0.73	0.70	0.12

PSU = perceived supervisor undermining; PCU = perceived coworkers undermining; SA = social adaptability; SCRD = self-control resource depletion; RMA = resource management ability.

**Table 2:** Descriptive statistics, internal consistency reliabilities, and correlation matrix.

Constructs	Mean	SD	1	2	3	4	5	6	7	8	9	10
(1) Gender (*T*1)	1.75	0.43										
(2) Age (*T*1)	28.77	4.01	−0.015									
(3) Job tenure (*T*1)	2.91	1.19	−0.016	0.051								
(4) Negative affect (*T*1)	3.44	1.15	−0.053	0.002	0.096^*∗*^	(0.93)						
(5) PSC (*T*1)	1.70	0.99	−0.157^*∗∗*^	−0.047	0.026	−0.017	(0.98)					
(6) PCU (*T*1)	1.63	0.95	−0.160^*∗∗*^	−0.064	−0.037	−0.023	0.831^*∗∗*^	(0.98)				
(7) SCRD (*T*1)	2.10	0.77	0.014	0.001	0.068	−0.077	0.372^*∗∗*^	0.335^*∗∗*^	(0.89)			
(8) SA (*T*2)	3.61	0.76	0.056	−0.011	0.044	0.009	−0.118^*∗*^	−0.115^*∗*^	−0.222^*∗∗*^	(0.94)		
(9) RMA (*T*2)	4.17	0.89	0.090	−0.006	−0.001	0.022	−0.163^*∗∗*^	−0.154^*∗∗*^	−0.102^*∗*^	0.499^*∗∗*^	(0.85)	
(10) KS (*T*3)	3.72	0.86	0.062	−0.009	0.066	0.011	−0.163^*∗∗*^	−0.171^*∗∗*^	−0.175^*∗∗*^	0.826^*∗∗*^	0.560^*∗∗*^	(0.94)

*N* = 440. Values of alpha are shown in parentheses. PSU = perceived supervisor undermining; PCU = perceived coworkers undermining; SA = social adaptability; SCRD = self-control resource depletion; RMA = resource management ability; KS = knowledge sharing; *T*1 = time 1; *T*2 = time 2; *T*3 = time 3; *T*4 = time 4; SCRD = self-control resource depletion. ^*∗*^*p*  <  0.05; ^*∗∗*^*p*  <  0.01; ^*∗∗∗*^*p*  <  0.001.

**Table 3:** Regression analyses.

	Self-control resource depletion			Knowledge sharing			
	*M*1	*M*2	*M*3	*M*1	*M*2	*M*3	*M*4
Gender	0.020	0.130	0.122	0.126	0.077	0.073	0.130
Age	0.030	0.004	0.005	−0.001	−0.003	−0.004	−0.001
Job tenure	0.050	0.057	0.059^∗^	0.047	0.044	0.043	0.057
Negative affect	−0.056	−0.050	−0.049	0.006	0.003	0.002	−0.006
PSU		0.300^*∗∗∗*^			−0.134^*∗∗*^		
PCU			0.285^*∗∗∗*^			−0.148^*∗∗*^	
SCRD							−0.202^*∗∗∗*^
*R * ^2^	0.012	0.157^*∗∗∗*^	0.130^*∗∗∗*^	0.008	0.032^*∗∗*^	0.034^*∗∗*^	0.041^*∗∗∗*^
∆*R*^2^		0.145^*∗∗∗*^	0.118^*∗∗∗*^		0.024^*∗∗*^	0.026^*∗∗*^	0.033^*∗∗∗*^

*N* = 440. Beta coefficients are unstandardized. ^*∗*^*p*  <  0.05; ^*∗∗*^*p*  <  0.01; ^*∗∗∗*^*p*  <  0.001. PSU = perceived supervisor undermining; PCU = perceived coworkers undermining; SCRD = self-control resource depletion.

**Table 4:** Bootstrap analysis of the direct and indirect effects.

Results of mediation analysis	Coefficient (SE)	LLCI	ULCI
*Direct and indirect effects of supervisor undermining on knowledge sharing*			
Total effect	−0.140^*∗∗*^ (0.406)	−0.220	−0.061
Direct effect	−0.098^*∗*^ (0.043)	−0.183	−0.012
Indirect effect	−0.043^*∗∗*^ (0.020)	−0.082	−0.003

*Direct and indirect effects of coworkers undermining on knowledge sharing*			
Total effect	−0.155^*∗∗*^ (0.043)	−0.239	−0.071
Direct effect	−0.115^*∗*^ (0.045)	−0.203	−0.026
Indirect effect	−0.040^*∗∗*^ (0.019)	−0.079	−0.005

*Moderating effect of social adaptability*			
*Direct and indirect effects of perceived supervisor undermining on knowledge sharing*			
Perceived supervisor undermining *X* social adaptability	−0.185^*∗∗∗*^ (0.044)	−0.272	−0.097
Direct effect	−0.098^*∗*^ (0.043)	−0.183	−0.012
−1SD ED-A fit (low level)	−0.061^*∗∗*^ (0.028)	−0.114	−0.004
1SD ED-A fit (mean level)	−0.041^*∗∗*^ (0.019)	−0.076	−0.003
+1SD ED-A fit (high level)	−0.020^*∗∗*^ (0.012)	−0.048	−0.001
Index of moderated mediation	0.027^*∗∗∗*^ (0.015)	0.001	0.057

*Direct and indirect effects of perceived coworkers undermining on knowledge sharing*			
Perceived coworkers undermining *X* social adaptability	−0.162^*∗∗*^ (0.048)	−0.256	−0.069
Direct effect	−0.115^*∗*^ (0.045)	−0.203	−0.026
−1SD ED-A fit (low level)	−0.056 (0.025)	−0.105	−0.007
1SD ED-A fit (mean level)	−0.038 (0.017)	−0.070	−0.005
+1SD ED-A fit (high level)	−0.019 (0.013)	−0.050	−0.000
Index of moderated mediation	0.024 (0.014)	0.001	0.053

*Moderating effect of resource management ability*			
*Conditional indirect effects of perceived supervisor undermining on knowledge sharing*			
Self-control resource depletion *X* resource management ability	−0.148^*∗∗*^ (0.049)	−0.244	−0.052
Direct effect	−0.045 (0.037)	−0.117	0.028
−1SD ED-A fit (low level)	−0.002 (0.021)	−0.041	0.044
1SD ED-A fit (mean level)	−0.036^*∗∗*^ (0.016)	−0.069	−0.005
+1SD ED-A fit (high level)	−0.071^*∗∗*^ (0.023)	−0.119	−0.028
Index of moderated mediation	−0.043^*∗∗*^ (0.018)	−0.078	−0.008

*Direct and indirect effects of perceived coworkers undermining on knowledge sharing*			
Self-control resource depletion *X* resource management ability	−0.151^*∗∗*^ (0.049)	−0.246	−0.055
Direct effect	−0.064 (0.038)	−0.138	0.011
−1SD ED-A fit (low level)	0.004 (0.020)	−0.038	0.041
1SD ED-A fit (mean level)	−0.033^*∗∗*^ (0.015)	−0.065	−0.005
+1SD ED-A fit (high level)	−0.067^*∗∗*^ (0.023)	−0.113	−0.026
Index of moderated mediation	−0.041^*∗∗*^ (0.017)	−0.075	−0.007

*N* = 440. Coefficients are unstandardized. LLCI = lower level of confidence interval; ULCI = upper level of confidence interval. ^*∗*^*p*  <  0.05; ^*∗∗*^*p*  <  0.01; ^*∗∗∗*^*p*  <  0.001.

## Data Availability

The data used to support the findings of this study are available from the corresponding authors upon reasonable request.
